# Randomized trial and multi-omics, machine learning–based mechanistic exploration of daixie decoction granules in type 2 diabetes

**DOI:** 10.3389/fphar.2025.1723584

**Published:** 2026-01-05

**Authors:** Zhong Zheng, Kepei Zhang, Xiaogang Ma, Yuezhou Qian, Miao Wang

**Affiliations:** Traditional Chinese Medicine Department, Longhua Hospital, Shanghai University of Traditional Chinese Medicine, Shanghai, China

**Keywords:** machine learning, multi-omics integration, network pharmacology, proteomics, traditional chinese medicine, type 2 diabetes mellitus

## Abstract

**Introduction:**

Traditional Chinese Medicine (TCM) offers multi-target strategies for Type 2 Diabetes Mellitus (T2DM), but its mechanisms are unclear. This study combined a randomized controlled trial (RCT) with a multi-omics approach to evaluate the efficacy of Daixie Decoction granules (DDG) as an add-on therapy to metformin and to generate mechanistic hypotheses using a multi-omics framework.

**Methods:**

We conducted a randomized, double-blind, placebo-controlled trial involving 136 randomized and 128 completed with DDG plus metformin or placebo plus metformin for 6 months. Mechanistic prediction was based on network pharmacology, integration of T2DM-related genes from public databases (GeneCards, DisGeNET, OMIM), and transcriptomic differentially expressed genes (DEGs) from GEO. Seven machine learning algorithms were applied to prioritize core targets from the overlapping candidate list. A nested serum proteomics sub-study within the randomized trial, with tissue-specific expression profiling (GTEx), was then used to explore the consistency of these computational predictions at the protein and tissue levels. Statistical analysis was performed using appropriate parametric and nonparametric tests, including ANCOVA where applicable.

**Results:**

DDG reduced HbA1c compared with placebo (−0.32%, P=0.032). Fasting plasma glucose showed a borderline reduction (P=0.050). Network pharmacology identified 617 potential targets intersecting with 2,652 DEGs, yielding 29 candidates. Using machine-learning combined with protein–protein interaction topology and literature support, we further prioritized eight core targets (P2RX7, IL1B, PTPN1, AKT2, CD38, NFE2L2, NOS3, and MERTK). Enrichment analyses of these candidates, together with serum proteomic profiling, implicated PI3K–Akt signaling, inflammatory and oxidative stress responses, and focal adhesion–related pathways.

**Conclusion:**

Clinically, DDG used as add-on therapy to metformin produced a modest but statistically significant improvement in glycemic control in patients with inadequately controlled T2DM. Our findings are consistent with the hypothesis that DDG may act through a multi-target network spanning inflammatory (P2RX7, IL1B), insulin/metabolic (PTPN1, AKT2, CD38), oxidative–endothelial (NFE2L2, NOS3) and vascular-resolution (MERTK) axes, generating testable mechanistic hypotheses for future experimental studies.

## 1 Introduction

Type 2 diabetes mellitus (T2DM) is a chronic, progressive metabolic disorder characterized by hyperglycemia resulting from insulin resistance and β-cell dysfunction (NCD Risk Factor Collaboration NCD-RisC, 2024). Its global prevalence continues to rise (Sun et al., 2022). While metformin remains the first-line therapy (ElSayed et al., 2023), many patients fail to achieve satisfactory glycemic control (Davies et al., 2022; Heyward et al., 2021). Hence, effective and safe adjunctive options are needed.

Traditional Chinese Medicine (TCM) is increasingly explored for metabolic disease (Chen et al., 2025; Liu et al., 2023). Compound herbal formulas, in particular, may modulate multiple pathological processes, aligning with T2DM´s multifactorial nature (Ni et al., 2024). Recent studies have shown promising effects of certain formulas in regulating glucose metabolism, inflammation, and oxidative stress (Meng et al., 2023; Zhang Q. et al., 2024). However, current research often focuses on isolated compounds or single mechanisms, neglecting the synergistic actions of whole formulas (Zhang B. et al., 2024). Yet integrative, translational multi-omics studies remain limited (Zhai et al., 2025).

The Daixie Decoction granules (DDG) is a classical TCM prescription for metabolic disorders. We first conducted a randomized controlled trial (RCT) to evaluate its efficacy on glycemic outcomes in patients with Type 2 Diabetes Mellitus (T2DM). An integrated multi-omics strategy-combining network pharmacology, transcriptomic analysis, machine learning, proteomics, and molecular docking-was then applied to elucidate its underlying mechanisms. This study is among the first to link randomized clinical evidence with multi-layer omics validation in a TCM formula for T2DM, offering novel mechanistic insights and translational value.

## 2 Methods

### 2.1 The workflow of the study

**FIGURE 1:**
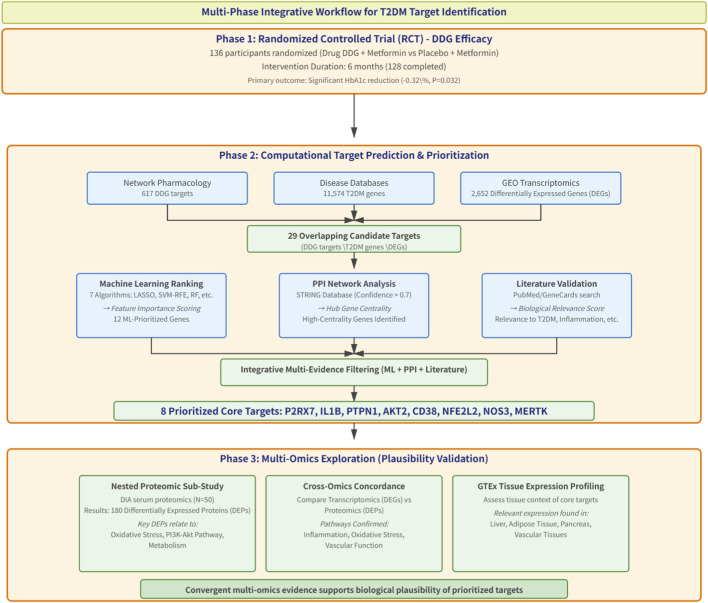
Workflow overview.

This study comprises three sequential phases: (1) clinical validation through a double-blind, randomized, placebo-controlled trial; (2) mechanistic prediction via network pharmacology,followed by machine-learning- based target prioritization; (3) a nested serum proteomics sub-study within the RCT, combined with tissue-specific expression profiling from GTEx, to explore molecular correlates of the intervention. The overall pipeline from clinical endpoints to computationally inferred mechanistic hypotheses is shown in Figure 1.

### 2.2 Randomized controlled trial (RCT)

#### 2.2.1 Study design and oversight

This was a prospective, two-arm, parallel-group randomized controlled trial (ChiCTR2000036290), conducted at Longhua Hospital, Shanghai University of Traditional Chinese Medicine, between September 2020 and March 2022, and was approved by the Ethics Committee of Longhua Hospital (2020LHSB026).

#### 2.2.2 Participants

From September 2020 to March 2022, 238 patients were screened, 136 participants were enrolled and randomized, and 128 were included in the final analysis.

Key inclusion criteria were as follows: (1) Age between 18 and 65 years; (2) Met the diagnostic criteria for type 2 diabetes; (3) New diagnosis of T2DM within the past 12 months with no prior use of any anti-diabetic medication; (4) A fasting plasma glucose (FPG) level between 7.0 mmol/L and 13.9 mmol/L after a 2-week run-in period of diet and exercise control.

Key Exclusion Criteria: (1) Glycated hemoglobin (HbA1c) level >9%; (2) Patients requiring insulin therapy; (3) Patients with severe hepatic insufficiency (transaminase levels more than twice the upper limit of normal), renal failure (serum creatinine >115 μmol/L), or heart failure (NYHA class III-IV); (4) Use of weight-loss supplements, antidepressants, or hormonal medications within the past 3 months or during the study period; (5) Pregnancy or lactation.

#### 2.2.3 Randomization and blinding

A centralized, stratified block randomization sequence was generated using SAS by an independent statistician. Allocation concealment was maintained using sequentially numbered opaque envelopes.

Double-blinding was ensured for all stakeholders. Placebo granules (identical in appearance and packaging) contained only excipients (sucrose, lactose, dextrin) and were supplied by the same manufacturer as DDG.

#### 2.2.4 Interventions

All participants received standardized guidance on diet and exercise. Participants were randomly assigned in a 1:1 ratio to either the intervention group or the control group for a 6-month treatment period.

Intervention Group (DDG + Metformin):

DDG granules: One 10 g packet, twice daily, dissolved in boiling water. Each packet of DDG consisted of the following herbal components: Atractylodes lancea 18 g, Atractylodes macrocephala 18 g, Salvia miltiorrhiza 15 g, Polygonum cuspidatum 12 g, Folium ilicis cornutae 15 g, Scutellaria 12 g, and Linderae radix 9 g. Metformin: Treatment was initiated at 0.5 g once daily after dinner for the first week. The dose was then titrated over 4 weeks based on patient tolerance to a maximum of 2 g daily (1 g twice daily) and maintained until the end of the treatment period.

Control Group (Placebo + Metformin):

Placebo granules: One packet, twice daily. The usage, dosage, and appearance were identical to the DDG granules.

Metformin: The dosage and titration schedule were identical to that of the intervention group.

The use of any other hypoglycemic, lipid-lowering, or weight-loss medications was prohibited during the trial.

#### 2.2.5 Outcomes and safety assessment

The primary endpoint of this study was the change in HbA1c from baseline to 6 months. Secondary endpoints included changes in FPG, fasting insulin, C-peptide levels, HOMA-IR and other related parameters. Safety was assessed by monitoring liver and renal function tests.

#### 2.2.6 Statistical analysis

Continuous variables were compared between groups using Student’s t-test; categorical variables were compared using the chi-square test. Normality of baseline variables was evaluated using the Shapiro–Wilk test and visual inspection of Q–Q plots. Given the sample size (N > 60), t-tests were considered robust even when minor deviations from normality were present.

Between-group differences in post-treatment outcomes were assessed using analysis of covariance (ANCOVA), with the corresponding baseline value included as a covariate. To address the modest baseline imbalance in BMI, additional post hoc sensitivity analyses were conducted using ANCOVA models further adjusted for baseline BMI. For skewed variables, log-transformed ANCOVA models were also performed as supplementary sensitivity analyses.

Missing data were handled using multiple imputation with predictive mean matching (PMM, m = 10), and estimates were pooled using Rubin’s rules. All analyses were two-sided with a significance threshold of P < 0.05.

### 2.3 Network pharmacology and bioinformatics analysis

#### 2.3.1 Identification of active compounds and potential targets

Active compounds in the DDG formula were retrieved from the Traditional Chinese Medicine Systems Pharmacology Database (TCMSP, http://tcmspw.com) (Ru et al., 2014) using predefined thresholds of oral bioavailability (OB) ≥ 30% and drug-likeness (DL) ≥ 0.18 (Xu et al., 2012). Compound–target associations were obtained from TCMSP or, if unavailable, predicted using SwissTargetPrediction (http://www.swisstargetprediction.ch/) (Gfeller et al., 2014). All targets were standardized via UniProt (https://www.uniprot.org) (UniProt Consortium, 2021), with species restricted to *Homo sapiens*.

#### 2.3.2 Identification of T2DM-associated disease targets

T2DM-related genes were collected from GeneCards (Stelzer et al., 2016), DisGeNET (Piñero et al., 2020), and OMIM (Amberger et al., 2019). Using the search term “Type 2 Diabetes Mellitus”. The union of all retrieved gene entries was compiled into a non-redundant disease target list.

#### 2.3.3 Differentially expressed genes (DEGs) analysis

Transcriptomic datasets from the Gene Expression Omnibus (GEO) database (Barrett et al., 2013) were integrated, that share the comparable biological contrast of T2DM versus healthy status. When merging multiple sets of transcriptome data, filtering low-abundance genes is a key preprocessing step. Specifically, genes with counts ≥10 in at least three samples are retained.

The merged count data was analyzed using the DESeq2 package (Love et al., 2014) in R (Team R. R, 2014). Batch effects originating from the distinct GEO series were controlled by incorporating a Batch factor into the design formula:{design} = {Batch} + {Group}. DEGs were identified using the DESeq2 with adjusted p-value <0.05 and |log2FC| ≥ 0.5 as cutoffs (Schurch et al., 2016), which is commonly used to balance sensitivity and biological relevance, avoiding overly stringent filters that may eliminate moderate but functionally meaningful changes.

#### 2.3.4 Identification of core therapeutic targets

We intersected: (1) DDG-predicted targets, (2) T2DM disease genes, and (3) GEO DEGs. To identify core therapeutic targets, we employed a three-step evidence strategy.

##### 2.3.4.1 Machine learning prioritization

The aim of the machine-learning analysis was to prioritize candidate genes according to their contribution to discriminating T2DM patients from non-diabetic controls. LASSO (Regression Shrinkage and Selection Via the Lasso, 1996), SVM-RFE (Guyon et al., 2002), random forest (RF) (Breiman, 2001), generalized linear model (GLM) (Nelder and Wedderburn, 1972), K-nearest neighbors (KNN) (Cover and Hart, 1967), neural network (NNET) (Rumelhart et al., 1986), and decision tree (DT) (Breiman et al, 2017)—were applied for target prioritization in R. The dependent variable was the binary T2DM status (T2DM vs. non-diabetic control), and the predictors were the expression levels of the overlapping candidate genes in the merged GEO expression matrix.

Samples were randomly split into a training set (70%) and an internal test set (30%), stratified by outcome. All models were trained using repeated 5-fold cross-validation (5 repeats) with hyperparameter tuning via grid search, using the area under the receiver operating characteristic curve (AUC) as the primary optimization metric. Model performance was summarized in both the cross-validation resamples and the test set using AUC, accuracy, sensitivity, specificity, and F1 score. For each algorithm, feature importance was extracted, and genes were ranked within each model. A consensus ranking was then obtained based on selection frequency and average importance ranking across the seven algorithms, and genes that consistently appeared among the top contributors were considered machine-learning- prioritized targets. Details of algorithm settings and tuning grids are provided in Supplementary Material 3.

##### 2.3.4.2 PPI analysis

Protein-protein interaction (PPI) analysis was conducted using the STRING database (version 12.0) with a confidence score >0.7. The resulting network was visualized in Cytoscape 3.10.0. Targets with high network centrality values were regarded as topological hubs.

##### 2.3.4.3 Literature-based and functional evidence

For each target, literature mining was performed using PubMed and GeneCards to evaluate biological relevance to T2DM, insulin resistance, oxidative stress, and inflammation.

Integrating all three evidence layers—machine learning importance, PPI hubness, and literature validation—candidates were assessed, and those meeting the multi-layered criteria were designated as core therapeutic targets for mechanistic interpretation and exploration.

#### 2.3.5 Functional enrichment analysis

GO and KEGG pathway enrichment analyses were conducted via DAVID (version 6.8) (Sherman et al., 2022), and results (p < 0.05) were visualized via Bioinformatics. com.cn.

### 2.4 Proteomic validation

The proteomic validation was conducted as a substudy within the RCT. Briefly, a subset of 25 participants from the DDG + metformin arm was randomly selected, and fasting serum samples were collected at baseline and at the 6-month visit. An additional group of age- and sex-matched healthy volunteers without diabetes (n = 25) was recruited as non-diabetic controls.

A data-independent acquisition (DIA)-based quantitative proteomic analysis was applied to evaluate the protein-level effects of the intervention (Ludwig et al., 2018). Sample preparation was conducted using the iST sample preparation kit (PreOmics, Germany) (PreOmics GmbH, 2023) according to the manufacturer’s protocol. Briefly, protein samples were lysed, denatured, reduced, alkylated, digested with trypsin, and desalted prior to LC-MS/MS analysis.

Peptides were analyzed using a timsTOF HT mass spectrometer (Meier et al., 2020) coupled with a nanoElute 2 LC system (Bruker Daltonik, Germany). A 20-min gradient was used for chromatographic separation on an AUR3-15075C18 analytical column (15 cm × 75 μm i. d., 1.7 μm particle size). DIA data were acquired in diaPASEF mode (Meier et al., 2018) with 24 * 25 Th isolation windows, covering an m/z range of 400–1000. Collision energy was ramped linearly during ion mobility scanning.

Raw data were processed using Spectronaut 18 (Biognosys, Switzerland) (Bruderer et al., 2015) with the human UniProt reference database. Peptide and protein FDR thresholds were set at 1% (Elias and Gygi, 2007). Quantification was prformed using the MaxLFQ algorithm (Cox et al., 2014). Differentially expressed proteins (DEPs) were defined as those with |fold change| > 1.5 and adjusted p-value <0.05 (Benjamini–Hochberg correction) (Benjamini and Hochberg, 1995). GO and KEGG enrichment analyses were conducted for DEPs.

To align with transcriptomic validation, downstream analyses emphasized the comparison between post-treatment T2DM and healthy controls.

### 2.5 Tissue-specific expression of core genes

The expression patterns of the identified core targets across human tissues were examined using data from the GTEx (https://www.gtexportal.org) (Consortium, 2020).

## 3 Results

### 3.1 Clinical efficacy of DDG in the randomized controlled trial (RCT)

**FIGURE 2:**
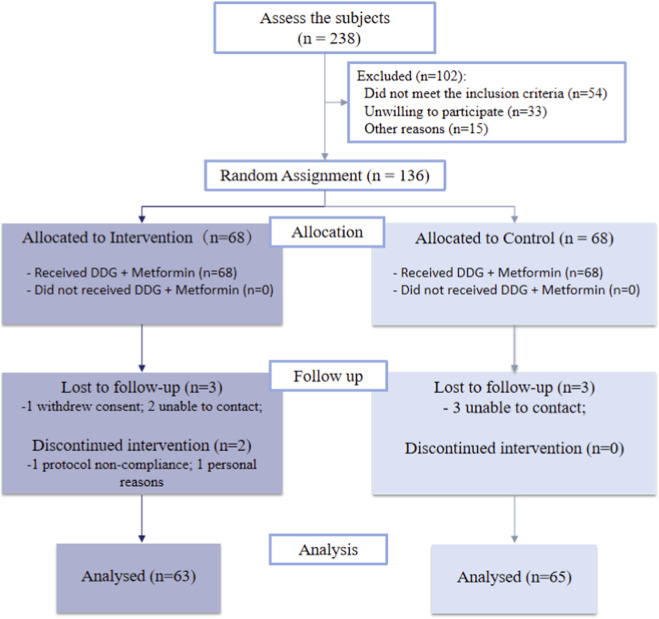
Participant flow through the study according to CONSORT.

A total of 136 participants were randomized (1:1), and 128 completed the study and were included in the final analysis (Figure 2). Data completeness was high, with missingness generally low (highest 12.1% in the treatment group). Missing values were handled using multiple imputation via predictive mean matching (m = 10). Sensitivity analyses,including complete-case analyses and log-transformed models for skewed variables, showed minimal impact on the key outcomes (Supplementary Figures S1–S3).

The assumption of normality of the baseline was formally tested using the Shapiro-Wilk test, and distributions were also visually inspected with Q-Q plots and histograms (Supplementary Table S1; Supplementary Figures S4-S5). Although some variables were not normally distributed according to the Shapiro-Wilk test, the t-test was considered robust due to the large sample size (N > 60) based on the Central Limit Theorem. Categorical variables were compared using the Chi-squared test. Baseline characteristics were assessed in the 128 participants contributing primary-outcome data. Most demographic and metabolic variable were broadly similar between groups (Table 1), indicating successful randomization. However, notable baseline imbalances were observed in BMI (28.38 vs. 27.29 kg/m^2^, P = 0.047).

**TABLE 1:** Baseline characteristics of participants.

Parameter	Treatment group (n = 63)	Control group (n = 65)	P-value
Demographics			
Age (years)	57.89 ± 10.37	55.17 ± 10.34	0.14^a^
Male sex, n (%)	38 (60.3)	41 (63.1)	0.889^b^
Metabolic parameters			
FPG (mmol/L)	7.13 ± 2.32	7.88 ± 2.95	0.111^a^
HbA1c (%)	7.46 ± 1.29	7.63 ± 1.95	0.576^a^
TG (mmol/L)	1.72 ± 1.05	1.59 ± 1.02	0.456^a^
TC (mmol/L)	4.06 ± 1.25	4.13 ± 1.49	0.782^a^
HDL-C (mmol/L)	1.07 ± 0.37	1.03 ± 0.32	0.489^a^
LDL-C (mmol/L)	2.3 ± 1.02	2.33 ± 1.11	0.875^a^
BMI (kg/m^2^)	28.38 ± 3.25	27.29 ± 2.91	0.047^a*^
Insulin metabolism			
C-peptide (ng/mL)	4.66 ± 4.52	4.25 ± 3.76	0.582^a^
Insulin (μU/mL)	12.54 ± 10.73	12.21 ± 9.1	0.851^a^
HOMA-IR	14.59 ± 12.24	16.07 ± 13.46	0.517^a^
Other parameters			
C-reactive protein (mg/L)	10.14 ± 18.68	8.55 ± 17.57	0.621^a^
ALT (U/L)	26.27 ± 21.66	24.28 ± 21.27	0.6^a^
AST (U/L)	23.23 ± 14.75	20.91 ± 19.44	0.449^a^
Creatinine (μmol/L)	65.98 ± 24.19	66.88 ± 22.77	0.829^a^

Data presentation: ^a^ Student’s t-test. ^b^ Chi-square test; Significance levels: *P < 0.05.

#### 3.1.1 Primary endpoint

After 6 months, the DDG group showed a significantly greater reduction in adjusted mean HbA1c compared to the control group (between-group difference: −0.32%, P = 0.032; Table 2).

**TABLE 2:** ANCOVA of end-of-study outcomes in the treatment and control groups.

Parameter	HbA1c < 7.5% subgroup			HbA1c ≥ 7.5% subgroup			Overall population		
	T	C	P-value	T	C	P-value	T	C	P-value
Metabolic parameters									
FPG (mmol/L)	5.91 ± 0.16	6.34 ± 0.15	0.056	6.09 ± 0.31	6.52 ± 0.31	0.341	5.98 ± 0.16	6.43 ± 0.16	0.05
HbA1c (%)	6.38 ± 0.11	6.49 ± 0.10	0.441	6.81 ± 0.19	7.38 ± 0.20	0.045*	6.56 ± 0.10	6.88 ± 0.10	0.032*
TG (mmol/L)	1.57 ± 0.09	1.62 ± 0.08	0.398	1.77 ± 0.12	1.54 ± 0.12	0.176	1.64 ± 0.07	1.58 ± 0.07	0.593
TC (mmol/L)	3.76 ± 0.14	3.50 ± 0.13	0.161	3.67 ± 0.19	3.67 ± 0.20	0.979	3.73 ± 0.11	3.56 ± 0.11	0.317
HDL-C (mmol/L)	1.01 ± 0.05	0.97 ± 0.04	0.528	1.04 ± 0.04	0.99 ± 0.04	0.392	1.02 ± 0.03	0.98 ± 0.03	0.444
LDL-C (mmol/L)	2.23 ± 0.08	2.12 ± 0.08	0.343	2.09 ± 0.11	2.25 ± 0.12	0.32	2.16 ± 0.07	2.17 ± 0.07	0.940
BMI (kg/m^2^)	27.63 ± 0.16	27.82 ± 0.15	0.388	26.65 ± 0.16	26.69 ± 0.16	0.856	27.19 ± 0.11	27.32 ± 0.11	0.412
Insulin metabolism									
C-peptide (ng/mL)	3.85 ± 0.41	3.65 ± 0.40	0.722	4.15 ± 0.46	3.84 ± 0.46	0.633	3.98 ± 0.31	3.74 ± 0.30	0.586
Insulin (μU/mL)	13.99 ± 1.23	13.10 ± 1.18	0.602	13.23 ± 0.82	10.56 ± 0.84	0.027*	13.63 ± 0.77	12.02 ± 0.76	0.141
HOMA-IR	14.25 ± 1.47	13.95 ± 1.40	0.886	13.72 ± 1.15	11.63 ± 1.17	0.21	14.02 ± 0.97	12.94 ± 0.95	0.425
Other parameters									
CRP (mg/L)	2.20 ± 0.33	2.49 ± 0.32	0.539	2.82 ± 0.69	3.80 ± 0.70	0.334	2.55 ± 0.36	3.0 ± 0.35	0.378
ALT (U/L)	21.62 ± 1.90	21.51 ± 1.82	0.968	25.85 ± 2.84	23.26 ± 2.89	0.524	23.54 ± 1.63	22.29 ± 1.61	0.587
AST (U/L)	23.86 ± 1.639	21.62 ± 1.57	0.331	24.58 ± 1.83	23.80 ± 1.86	0.766	24.33 ± 1.22	22.42 ± 1.20	0.268
Creatinine (μmol/L)	66.96 ± 2.62	63.98 ± 2.51	0.415	68.84 ± 12.50	82.29 ± 12.7	0.459	67.62 ± 5.75	72.07 ± 5.66	0.582

Data presentation: All between-group comparisons were adjusted for baseline values using ANCOVA., Significance levels: *P < 0.05.

As an exploratory analysis, we further stratified participants by baseline HbA1c of 7.5%, a threshold commonly used to indicate suboptimal glycemic control in clinical practice. In the subgroup, the benefit was primarily driven by participants with a baseline HbA1c ≥ 7.5% (P = 0.045), whereas no significant effect was observed in the <7.5% stratum (P = 0.441).

#### 3.1.2 Secondary endpoints

Fasting plasma glucose (FPG) showed a borderline reduction favoring the treatment group (P = 0.050; Table 2).

There were no significant overall between-group differences in HOMA-IR, C-peptide, or fasting insulin. In the same exploratory stratification by baseline HbA1c ≥ 7.5% subgroup, fasting insulin levels were significantly higher in the DDG group (P = 0.027), suggesting a potential improvement in β-cell function in patients with poor baseline control. Other metabolic markers, including lipids, BMI, and CRP, did not differ significantly between groups.

Log-transformed sensitivity analyses yielded results consistent with the primary analysis (Supplementary Tables S2, S3), supporting the robustness of the findings.

#### 3.1.3 Safety

No hypoglycemic episodes were observed, and no intervention-related SAEs occurred. Occasional, mild, self-limiting diarrhea was reported in both groups; a contribution from metformin and/or dietary factors cannot be excluded. Routine safety laboratories (hepatic/renal) remained overall stable without clinically meaningful abnormalities.

#### 3.1.4 Baseline-adjusted sensitivity analysis

Given the modest baseline imbalance in BMI between groups, we performed post hoc sensitivity analyses using ANCOVA models further adjusted for baseline BMI. In these BMI-adjusted models, the between-group differences in HbA1c and FPG remained similar in magnitude to the primary analyses (BMI-adjusted LS means 6.58% vs. 6.87% for HbA1c and 5.98 vs. 6.43 mmol/L for FPG in the DDG and control groups, respectively), although the P values became borderline (P = 0.053 and P = 0.052, Supplementary Table S4). No between-group differences were observed for HOMA-IR, lipid parameters, CRP, liver enzymes or creatinine after additional BMI adjustment.

### 3.2 Prediction of core targets

#### 3.2.1 Identification of potential therapeutic targets

**FIGURE 3:**
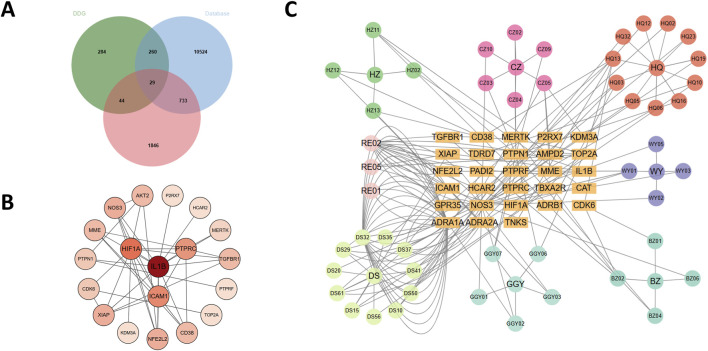
Analysis of DDG targets in T2DM. **(A)** Venn diagram of 29 core targets from compound, disease, and DEG overlap. **(B)** PPI network of candidate targets. **(C)** Herb–compound–target network.

We first retrieved a total of 144 active compounds and 617 corresponding drug targets from the TCMSP database, which includes the seven herbal components of DDG. Concurrently, we collected 11,574 T2DM-related genes from three public disease databases: GeneCards, DisGeNET, and OMIM. Differential gene expression analysis was then performed by integrating the GEO datasets GSE137317, GSE153315, and GSE153792, yielding 12,353 genes. After Batch effects originating, we apply the thresholds of adjusted p-value (padj) < 0.05 and |log2FoldChange| ≥ 0.5, we identified 2,652 differentially expressed genes (DEGs). By intersecting the drug targets, disease-related genes, and DEGs, we obtained 29 overlapping candidate targets, which were considered potential therapeutic targets through which DDG may exert its effects on T2DM (Figure 3A). The full lists of drug targets, disease-related genes, DEGs, and overlapping candidate targets are provided in Supplementary Material 2 (Excel), with each dataset presented in a separate sheet.

#### 3.2.2 Construction of protein–protein interaction and herb–compound–target networks

The 29 intersected candidate genes were imported into the STRING database to construct a PPI network, which was subsequently visualized using Cytoscape (Figure 3B). The resulting network revealed significant interactions among the targets, with IL1B, HIF1A, PTPRC, NOS3, AKT2, CD38, NFE2L2 and ICAM1 identified as central hub genes due to their high degree of connectivity. We further constructed a herb–compound–target network based on the seven herbs, 144 active compounds, and the 29 candidate targets (Figure 3C). Among these, scutellaria baicalensis (Huang Qin) and radix salviae (Dan Shen) contained the greatest number of active compounds and regulated the largest number of targets. Notably, dan-shexinkum d (PubChem CID: 124307626) was identified as the most central compound, targeting 21 genes, followed by quercetin (PubChem CID: 5280343) with 17 targets.

#### 3.2.3 Machine-learning–based prioritization and integrative definition of core targets

**FIGURE 4:**
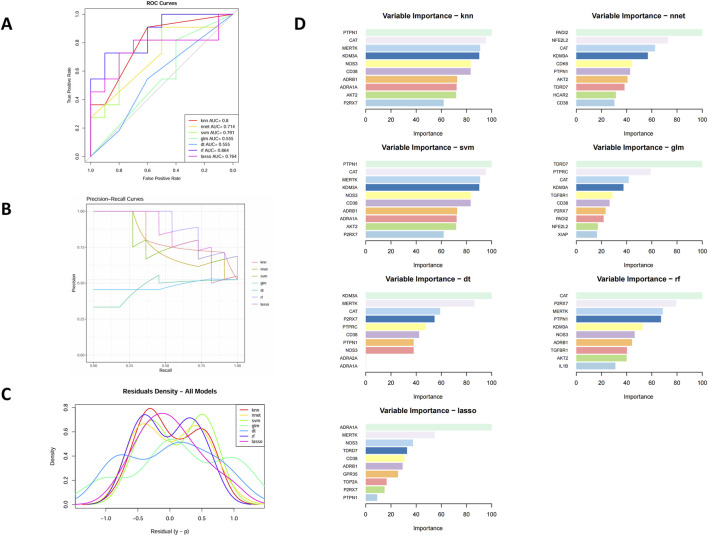
Evaluation of machine learning models for core gene selection. **(A)** ROC curves of seven algorithms. **(B)** Precision–recall curves. **(C)** Residual histograms. **(D)** Top 10 feature genes ranked by model importance.

To further prioritize the 29 overlapping candidate genes, we applied seven supervised machine-learning algorithms (LASSO, SVM-RFE, RF, GLM, KNN, NNET, and DT) using T2DM status (T2DM vs. control) as the dependent variable and the expression levels of the 29 genes as predictors. As detailed in Section 2.3.4, all models were trained with repeated 5-fold cross-validation (5 repeats), and their performance in both the cross-validation resamples and the internal test set was summarized by AUC(Figure 4A), accuracy, sensitivity, specificity, and F1 score (Supplementary Table S1). The ROC and precision–recall curves (Figures 4A,B) showed that all seven models captured discriminative signal from the 29-gene panel, supporting their use as feature-ranking tools. Residual diagnostics (Figure 4C) did not reveal major systematic bias, with residuals approximately centered around zero and comparable dispersion across models.

We next focused on the feature-importance patterns generated by these models. Across the seven algorithms, several genes—including P2RX7, PTPN1, CD38, KDM3A, CAT, NOS3, MERTK, and AKT2—repeatedly appeared among the top-ranked features, suggesting robust and model-independent contributions to the T2DM–control separation (Figure 4D). When these ML-derived importance rankings were compared with the PPI network structure, we observed considerable overlap: for example, NOS3, AKT2, CD38, and NFE2L2 were not only highly ranked by multiple ML models but also occupied central positions in the PPI network (Figure 3B).

To define a focused set of core therapeutic targets, we integrated three layers of evidence: (1) high and consistent feature importance across multiple ML algorithms, (2) high connectivity (hub-like behavior) in the PPI network, and (3) strong prior experimental or clinical evidence linking each gene to T2DM, metabolic dysregulation, and inflammation/oxidative stress.

Through this integrative filtering, we prioritized a set of key targets, including P2RX7 and IL1B (inflammasome- and NLRP3-related inflammatory axis), PTPN1, AKT2, and CD38 (insulin resistance and metabolic signaling axis), NFE2L2 and NOS3 (oxidative-stress and endothelial-protective axis), and MERTK (inflammation-resolution and plaque-stabilizing axis). These genes were carried forward as core therapeutic targets of DDG in subsequent mechanistic analyses. The main literature supporting the involvement of each target in T2DM is summarized in Supplementary Table S2.

### 3.3 Multi-omics exploration of putative mechanisms

To explore whether the computationally prioritized targets and pathways are consistent with independent data sources, we conducted a series of exploratory evaluation analyses. These included (1) quantitative proteomic profiling to confirm protein-level alterations, (2) tissue-specific expression profiling to assess biological plausibility, and (3) molecular docking simulations to evaluate the binding potential between DDG-derived compounds and representative targets.

#### 3.3.1 Proteomic profiling in the nested RCT sub-cohort

**FIGURE 5:**
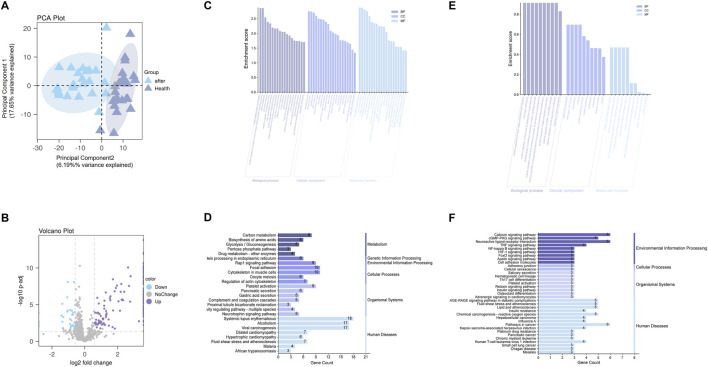
Proteomic and transcriptomic analyses. **(A)** PCA plot showing separation between post-treatment T2DM samples and healthy controls; the percentages in the axis labels denote the variance explained by each component (effect size). **(B)** Volcano plot of differentially expressed proteins; the x-axis displays the effect size (log2 fold change), and the y-axis shows statistical significance (–log10 adjusted p value). **(C,E)** GO enrichment analyses for proteomic DEPs **(C)** and transcriptomic candidate genes **(E)**; bar length represents the enrichment effect size (enrichment score), with terms grouped by Biological Process (BP), Cellular Component (CC), and Molecular Function (MF). **(D,F)** KEGG enrichment analyses for proteomic DEPs **(D)** and transcriptomic candidates **(F)**; bar length indicates the effect size, defined as the number of genes enriched in each pathway (Gene Count).

A total of 920 protein groups were identified across the serum samples. Between the post-treatment T2DM group and healthy controls, 180 proteins were differentially expressed based on the criteria of |fold change| > 1.5 and adjusted p-value <0.05, including 146 upregulated and 34 downregulated proteins. PCA revealed clear clustering between the two groups, indicating a distinct protein expression profile (Figure 5A). The volcano plot further illustrated the distribution of DEPs in terms of log2 fold change and statistical significance (Figure 5B). The complete differential expression matrix for proteomics is provided in Supplementary Material 4 (Excel).

GO enrichment analysis (Figure 5C) revealed that the DEPs were enriched in biological processes such as oxidative stress response, insulin signaling (e.g., “positive regulation of protein kinase B signaling”), glycolysis/gluconeogenesis, and protein deubiquitination. In terms of cellular components, they were mainly localized to cytoplasmic vesicles, secretory granules, extracellular matrix, and platelet-related structures. Molecular functions were associated with protein binding, oxidoreductase activity, and cytoskeletal structural components.

KEGG pathway enrichment (Figure 5D) indicated that DEPs were primarily involved in metabolic pathways such as carbon metabolism, amino acid biosynthesis, glycolysis/gluconeogenesis. Significant enrichment was also found in pathways related to platelet activation, focal adhesion, pancreatic secretion, and the complement and coagulation cascades. Morever, the “fluid shear stress and atherosclerosis” pathway was highlighted.

#### 3.3.2 Cross-omics pathway concordance between transcriptomic candidates and proteomic DEPs

To assess cross-omics pathway consistency, we compared enrichment profiles of 29 GEO-derived transcriptomic targets and 180 serum DEPs.

GO and KEGG (Figures 5E,F) analyses of the 29 transcriptomic candidate genes highlighted several key biological programs, including inflammatory and immune signaling (“cytokine–cytokine receptor interaction”, “NF-κB signaling”), insulin-related metabolic regulation (“PI3K–Akt signaling pathway”), oxidative stress responses, and cytoskeletal remodeling and adhesion (“focal adhesion”, “regulation of actin cytoskeleton”).

Strikingly, these pathway themes were independently recapitulated in the proteomic DEPs. Proteomic enrichment revealed oxidative stress–related processes, PI3K–Akt signaling, glycolysis/gluconeogenesis, complement and coagulation cascades, platelet activation, and the “fluid shear stress and atherosclerosis” pathway—overlapping with the transcriptomic signatures of vascular regulation and cytoskeletal organization.

Together, this cross-omics concordance indicates that similar inflammatory, metabolic, oxidative stress–related, and vascular/cytoskeletal pathways are enriched at both the transcriptomic and proteomic levels. These observations suggest that the biological processes highlighted in the candidate-target analysis are reproducible across omics layers.

#### 3.3.3 Tissue expression profiles of core target genes

To explore the tissue context and biological plausibility of the eight prioritized targets, we retrieved transcripts-per-million (TPM) expression data for the core genes from the GTEx database.

**FIGURE 6:**
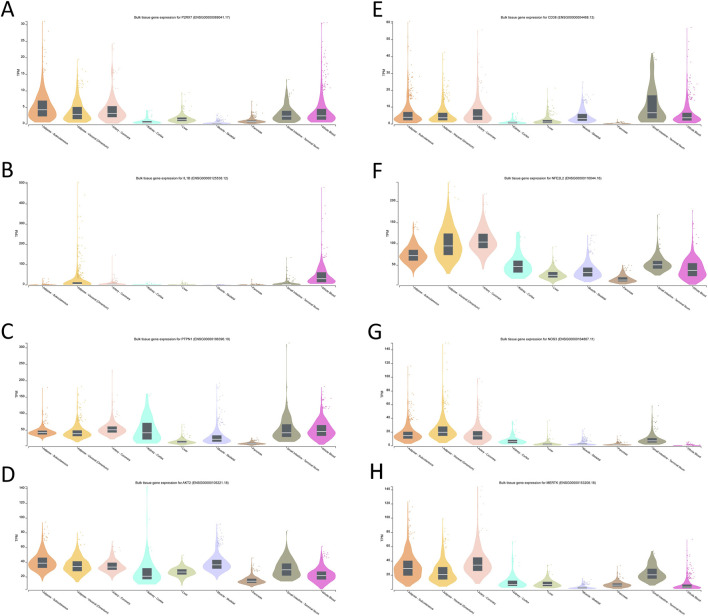
Tissue-specific expression profiles of the core target genes.**(A)** P2RX7; **(B)** IL1B; **(C)** PTPN1; **(D)** AKT2; **(E)** CD38; **(F)** NFE2L2; **(G)** NOS3; **(H)** MERTK.

For P2RX7, IL1B, PTPN1, AKT2, CD38, NFE2L2, NOS3 and MERTK, GTEx profiles showed higher expression in metabolically and vasculature-relevant tissues, including adipose tissue, liver, skeletal muscle, arterial segments and whole blood (Figures 6A-G). These patterns indicate that the multi-omics–derived targets are preferentially expressed in organs and cell types implicated in insulin resistance, vascular dysfunction and chronic inflammation, supporting their biological relevance to T2DM.

## 4 Discussion

### 4.1 Clinical efficacy of DDG

Our trial demonstrates that DDG provides a modest but statistically significant reduction in HbA1c, particularly for patients with poorer glycemic control who are most in need of adjunctive therapies. The observed increase in fasting insulin within this subgroup, despite no overall change in HOMA-IR, could be consistent with enhanced insulin secretion, but in the absence of stimulated insulin or C-peptide measurements and given the unchanged HOMA-IR, this should not be interpreted as direct evidence of β-cell functional improvement. Nonetheless, as subgroup analyses were exploratory and the overall insulin comparison was null, this finding should be interpreted cautiously and viewed as hypothesis-generating.

Other metabolic markers, including C-peptide, lipids, and CRP, showed no significant between-group differences over 6 months. Given that CRP represents only a crude measure of systemic inflammation, these findings do not exclude more subtle anti-inflammatory or antioxidant effects, which are warrant further mechanistic investigation.

### 4.2 Exploratory, multi-omics interpretation of the core DDG targets

#### 4.2.1 Hypothesis-generating mechanistic axes suggested by the core targets

##### 4.2.1.1 P2RX7–IL1B: inflammasome-related inflammatory axis

P2RX7 and IL1B were jointly prioritized by the integrative filtering and cluster in a classical NLRP3–inflammasome pathway (Meier et al., 2025; Kong et al., 2022). Prior experimental and clinical work has already linked P2X7 signaling and IL-1β production to low-grade inflammation, β-cell stress and vascular injury in T2DM (Wang et al., 2020; Sun et al., 2012), so their emergence here is consistent with a contributory inflammatory background on which DDG was tested, and further targeted experiments are needed to determine whether the formula additionally exerts a specific anti-inflammasome action.

##### 4.2.1.2 PTPN1–AKT2–CD38: insulin signaling and glucose-metabolic axis

PTPN1, AKT2 and CD38 are all involved in insulin receptor signaling, downstream Akt activation, and β-cell/NAD^+^-dependent metabolic regulation (Florez et al., 2005; Escande et al., 2013; Cho et al., 2001). These genes have been implicated in insulin resistance and glycemic control in previous studies, and their joint prioritization in insulin-responsive tissues provides a plausible, though still indirect, link between the modest HbA1c improvement observed in our trial and potential effects of DDG on insulin-related pathways.

##### 4.2.1.3 NFE2L2–NOS3: oxidative-stress and endothelial axis

NFE2L2 (Nrf2) coordinates antioxidant responses (Wu et al., 2020; David et al., 2017), whereas NOS3 (eNOS) is a key determinant of endothelial nitric oxide bioavailability and vascular homeostasis (Janaszak-Jasiecka et al., 2023). Both have been associated with oxidative stress, endothelial dysfunction and diabetic vascular complications, so their appearance among the core targets fits with a working hypothesis that DDG may influence redox and endothelial biology, in line with—but not conclusively demonstrated by—the oxidative/vascular pathways enriched in our omics analyses.

##### 4.2.1.4 MERTK: inflammation-resolution and plaque-stabilizing axis

MERTK has been linked to more stable atherosclerotic plaque phenotypes (Cai et al., 2017). Its selection as a core target, together with the enrichment of extracellular matrix, complement and coagulation-related pathways, suggests a possible connection between DDG and vascular remodeling or thrombo-inflammatory processes, which remains to be tested in studies with appropriate vascular endpoints.

Taken together, these four axes provide a conceptual organization of the core targets that is compatible with existing literature on T2DM pathophysiology, but they should be regarded as hypothesis-generating rather than mechanistically definitive.

#### 4.2.2 Consistency between transcriptomic targets and serum proteomics

The pathway enrichment results from serum proteomics showed a similar pattern to the biological themes suggested by the transcriptomic candidate targets. Although the core genes were not always directly detected at the protein level, the proteomic DEPs were enriched for immune/inflammatory processes, metabolism-related pathways and vascular/ECM remodeling, which correspond to the inflammatory (P2RX7–IL1B), insulin/metabolic (PTPN1–AKT2–CD38), oxidative/endothelial (NFE2L2–NOS3) and pro-resolving (MERTK) axes described above. We therefore regard this agreement at the pathway level as supportive that the prioritized targets reflect biologically relevant programs in T2DM, while acknowledging that the present data cannot link individual genes to specific protein changes or separate direct from downstream effects of DDG.

#### 4.2.3 Tissue expression context from GTEx and remaining uncertainties

GTEx analysis places the core targets in a tissue context that is compatible with their putative roles in T2DM: several genes show appreciable expression in liver, adipose tissue, skeletal muscle and pancreas, as well as in vascular and immune-rich tissues, in line with involvement in insulin resistance, low-grade inflammation and vascular dysfunction. However, GTEx is based on non-diabetic donors and bulk tissues, and thus cannot capture disease-specific regulation, cellular heterogeneity or any pharmacological modulation by DDG. Accordingly, the GTEx findings mainly provide biological plausibility and anatomical context for the highlighted axes, and future work using patient-derived, single-cell or spatial datasets will be needed to refine these hypotheses.

### 4.3 Study limitations and future directions

Several limitations should be acknowledged. Clinically, the modest sample size and trial duration limit conclusions regarding long-term durability and diabetic complications. Although sensitivity analysis adjusting for baseline BMI showed consistent effect sizes for glycemic improvement, the observed imbalance in baseline BMI between groups may have contributed to the strength of the statistical significance in the primary analysis. Future studies should ensure balanced randomization or consider stratification by BMI to confirm these findings. Furthermore, the absence of specific biomarkers for oxidative or inflammatory pathways in the RCT prevents direct confirmation of the proposed axes. Mechanistically, our findings rely on integrative bioinformatics; while these rigorous computational approaches prioritize targets, they remain hypothesis-generating and lack direct experimental validation.

Future research should focus on: (1) mechanistic validation through targeted gene knockout/overexpression studies; (2) identification and isolation of specific active compounds responsible for observed effects; (3) larger-scale clinical trials with extended follow-up; and (4) investigation of optimal dosing regimens and patient selection criteria.

## 5 Conclusion

In this randomized trial, adding DDG to metformin yielded a modest improvement in HbA1c, particularly in patients with higher baseline levels, without inducing broader short-term metabolic remodeling. While the clinical magnitude was limited, these findings provided a crucial phenotypic anchor for our multi-omics analysis, which mapped the observed effects to a prioritized network of eight core targets (e.g., P2RX7, AKT2, NFE2L2) spanning inflammatory, metabolic, and vascular axes. Collectively, this study offers preliminary support for DDG as an adjunctive option and, more importantly, validates a strategy for decoding complex herbal interventions by bridging empirical clinical signals with data-driven mechanistic hypotheses.

## Data Availability

The proteomics data has been deposited in the iProX repository, accession number PXD069763 (Project ID IPX0013864002). The processed datasets supporting the findings of this study are available in the Supplementary Materials. Further requests can be directed to the corresponding authors.

## Glossary

AKT2: AKT Serine/Threonine Kinase 2
ALT: Alanine aminotransferase
ANCOVA: Analysis of covariance
AST: Aspartate aminotransferase
ATP: Adenosine triphosphate
AUC: Area under the curve
BMI: Body mass index
CD38: Cluster of Differentiation 38
CRP: C-reactive protein
DDG: Daixie Decoction granules
DEGs: Differentially expressed genes
DEPs: Differentially expressed proteins
DIA: Data-independent acquisition
DisGeNET: Database of gene-disease associations
DL: Drug-likeness
DT: Decision tree
FDR: False discovery rate
FPG: Fasting plasma glucose
GEO: Gene Expression Omnibus
GLM: Generalized linear model
GO: Gene Ontology
GTEx: Genotype-Tissue Expression
HbA1c: Glycated hemoglobin
HDL-C: High-density lipoprotein cholesterol
HOMA-IR: Homeostatic Model Assessment for Insulin Resistance
IL1B: Interleukin 1 Beta
KEGG: Kyoto Encyclopedia of Genes and Genomes
KNN: K-nearest neighbors
LASSO: Least absolute shrinkage and selection operator
LC-MS/MS: Liquid chromatography-tandem mass spectrometry
LDL-C: Low-density lipoprotein cholesterol
MERTK: MER Tyrosine Kinase
NLRP3: NLR family pyrin domain containing 3
NNET: Neural network
NFE2L2: Nuclear factor erythroid 2-related factor 2
NOS3: Nitric Oxide Synthase 3 (eNOS)
NYHA: New York Heart Association
OB: Oral bioavailability
OMIM: Online Mendelian Inheritance in Man
P2RX7: P2X purinoceptor 7
PCA: Principal component analysis
PMM: Predictive mean matching
PTPN1: Protein Tyrosine Phosphatase Non-Receptor Type 1
PPI: Protein-protein interaction
RCT: Randomized controlled trial
RF: Random forest
ROC: Receiver operating characteristic
SVM-RFE: Support vector machine-recursive feature elimination
T2DM: Type 2 diabetes mellitus
TC: Total cholesterol
TCM: Traditional Chinese Medicine
TCMSP: Traditional Chinese Medicine Systems Pharmacology Database
TG: Triglycerides
TPM: Transcript per million
