# Supplementary material for: Randomized trial and multi-omics, machine learning–based mechanistic exploration of daixie decoction granules in type 2 diabetes
Source: Front Pharmacol. 2026 Jan 5;16:1723584. doi: 10.3389/fphar.2025.1723584 (PMC12812742; doi:10.3389/fphar.2025.1723584)
Supplement: Supplementary file 1 [file DataSheet1.zip › Supplementary Material1.docx]

# Supplementary Material 1

This document contains supplementary tables and figures supporting the imputation diagnostics and regression model stability analyses described in the main text.

## Table S1. Normality and Homogeneity of Variance of Baseline Data.

| **Variable** | **Shapiro_W** | **Shapiro_p** | **Normality** | **Levene_F** | **Levene_p** | **Equal_Variance** |
| --- | --- | --- | --- | --- | --- | --- |
| Age | 0.079 | 0.779 | Yes | 0.727 | <0.001 | No |
| Gender | 0.221 | 0.639 | Yes | 0.609 | <0.001 | No |
| FPG | <0.001 | 0.991 | Yes | 0.982 | 0.448 | Yes |
| HbA1c | 0.232 | 0.631 | Yes | 0.945 | 0.003 | No |
| CP | 0.154 | 0.696 | Yes | 0.831 | <0.001 | No |
| INS | 0.204 | 0.653 | Yes | 0.507 | <0.001 | No |
| HOMAIR | 0.006 | 0.937 | Yes | 0.931 | <0.001 | No |
| BMI | 2.554 | 0.113 | Yes | 0.882 | <0.001 | No |
| TC | 0.102 | 0.75 | Yes | 0.616 | <0.001 | No |
| TG | 1.644 | 0.202 | Yes | 0.928 | <0.001 | No |
| LDLC | 0.896 | 0.346 | Yes | 0.83 | <0.001 | No |
| HDLC | 0.617 | 0.434 | Yes | 0.861 | <0.001 | No |
| CRP | 0.021 | 0.884 | Yes | 0.84 | <0.001 | No |
| ALT | 0.14 | 0.709 | Yes | 0.946 | 0.001 | No |
| AST | 0.238 | 0.627 | Yes | 0.942 | <0.001 | No |
| Cr | 0.12 | 0.73 | Yes | 0.805 | <0.001 | No |

## Table S2. Residual Assumption Tests for ANCOVA.

| Variable | HbA1c < 7.5% Subgroup | | HbA1c ≥ 7.5% Subgroup | | Overall Population | |
| --- | --- | --- | --- | --- | --- | --- |
|  | shapiro_p | levene_p | shapiro_p | levene_p | shapiro_p | levene_p |
| BMI | <0.001 | 0.161 | <0.001 | 0.409 | <0.001 | 0.103 |
| TG | 0.002 | 0.519 | 0.524 | 0.786 | 0.001 | 0.752 |
| TC | 0.59 | 0.737 | 0.4 | 0.075 | 0.662 | 0.097 |
| HDLC | 0.648 | 0.999 | 0.045 | 0.168 | 0.237 | 0.333 |
| LDLC | 0.621 | 0.086 | 0.003 | 0.694 | 0.001 | 0.185 |
| FPG | 0.059 | 0.813 | <0.001 | 0.076 | <0.001 | 0.118 |
| HbA1c | 0.305 | 0.126 | <0.001 | 0.143 | <0.001 | 0.772 |
| CP | <0.001 | 0.657 | 0.004 | 0.744 | <0.001 | 0.885 |
| INS | <0.001 | 0.572 | 0.65 | 0.048 | <0.001 | 0.742 |
| HOMAIR | <0.001 | 0.726 | 0.074 | 0.111 | <0.001 | 0.54 |
| CRP | <0.001 | 0.738 | <0.001 | 0.224 | <0.001 | 0.554 |
| ALT | 0.15 | 0.643 | <0.001 | 0.347 | <0.001 | 0.58 |
| AST | 0.017 | 0.922 | 0.04 | 0.827 | 0.002 | 0.79 |
| Cr | 0.174 | 0.393 | <0.001 | 0.33 | <0.001 | 0.248 |

## Table S3. Sensitivity analyses of log-transformed outcomes (overall and by HbA1c subgroup).

| Variable | Subgroup | Estimate/Diff | SE | P-value |
| --- | --- | --- | --- | --- |
| TG | Overall | -0.054 | 0.102 | 0.6 |
| LDLC | Overall | 0.007 | 0.098 | 0.941 |
| TG | Low | 0.061019 | 0.078083 | 0.437247 |
| AST | Low | -0.09962 | 0.103596 | 0.339638 |
| CR | Low | -0.036 | 0.054891 | 0.514151 |
| HDLC | High | -0.06325 | 0.056578 | 0.268555 |
| LDLC | High | 0.114602 | 0.081549 | 0.165657 |
| FPG | High | 0.054458 | 0.067588 | 0.423933 |
| CP | High | -0.07503 | 0.201463 | 0.711044 |
| AST | High | -0.00358 | 0.10608 | 0.973216 |

## Sensitivity analyses confirmed that results for TG, LDL-C and other subgroup variables remained consistent with the primary ANCOVA findings, indicating robustness of the conclusions.

## Table S4. Sensitivity analyses of ANCOVA of End-of-Study Outcomes Adjusted for Baseline BMI.

| Parameter | Overall Population | | | | |
| --- | --- | --- | --- | --- | --- |
|  | T | | C | | P-value |
| Metabolic Parameters |  |  | |  |  |
| FPG (mmol/L) | 5.98±0.16 | | 6.43±0.16 | | 0.052 |
| HbA1c (%) | 6.58±0.10 | | 6.87±0.10 | | 0.053 |
| TG (mmol/L) | 1.63±0.07 | | 1.58±0.07 | | 0.661 |
| TC (mmol/L) | 3.73±0.11 | | 3.56±0.11 | | 0.303 |
| HDL-C (mmol/L) | 1.01±0.03 | | 0.99±0.03 | | 0.597 |
| LDL-C (mmol/L) | 2.17±0.07 | | 2.16±0.07 | | 0.946 |
| BMI (kg/m²) | 27.19±0.11 | | 27.32±0.11 | | 0.412 |
| Insulin Metabolism |  |  | |  |  |
| C-peptide (ng/mL) | 4.00±0.31 | | 3.72±0.31 | | 0.537 |
| Insulin (μU/mL) | 13.42±0.77 | | 12.22±0.76 | | 0.273 |
| HOMA-IR | 13.76±0.96 | | 13.19±0.95 | | 0.679 |
| Other Parameters |  |  | |  |  |
| CRP (mg/L) | 2.65±0.36 | | 2.90±0.35 | | 0.620 |
| ALT (U/L) | 23.47±1.65 | | 22.36±1.63 | | 0.637 |
| AST (U/L) | 24.22±1.23 | | 22.53±1.21 | | 0.333 |
| Creatinine (μmol/L) | 67.66±5.82 | | 72.03±5.72 | | 0.597 |

Data presentation: All between-group comparisons were adjusted for baseline BMI using ANCOVA. Significance levels: P < 0.05.

## Figure S1.Missing-data pattern before multiple imputation.


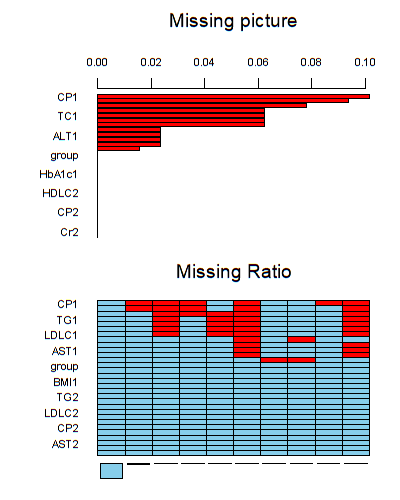


Figure S1 presents the proportion of missing data for each variable in the dataset. The missingness was mostly confined to a limited number of variables, and no systematic patterns were observed, consistent with the MAR assumption.

## Figure S2. Density distribution of imputed vs observed data (pwm, m=10).


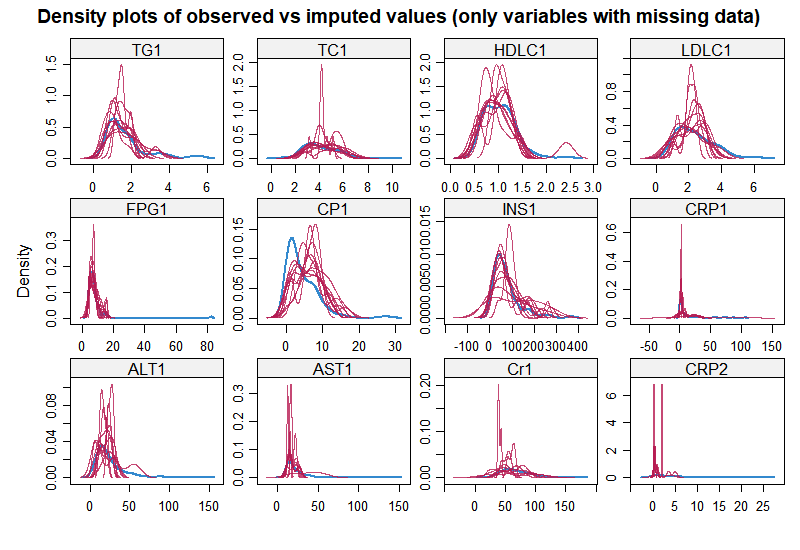


Figure S2 shows that the imputed values followed the distribution of observed data, suggesting that the imputation preserved the original data structure.

## Figure S3. Dot plot of pooled estimates across 10 imputations (pwm, m=10).


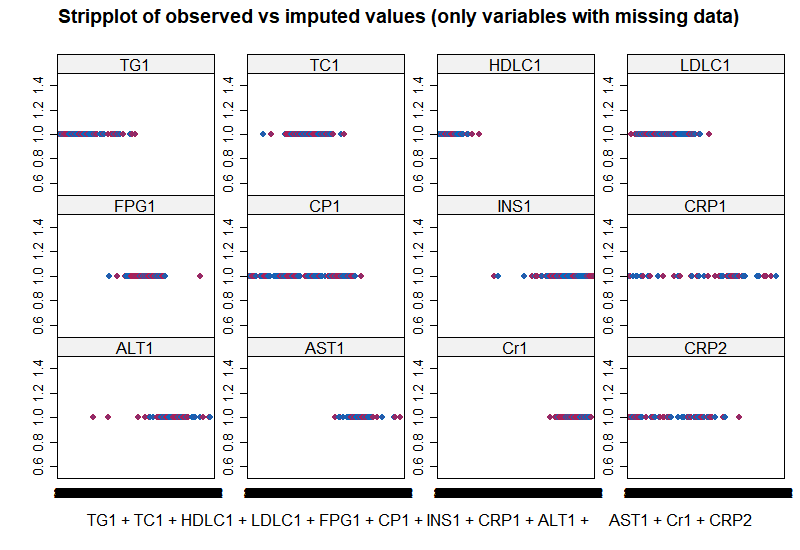


Each dot represents the estimate from a single imputed dataset, with minimal variation across imputations, indicating robustness of the results.

## Figure S4. Distribution of baseline variables (histograms with density curves).


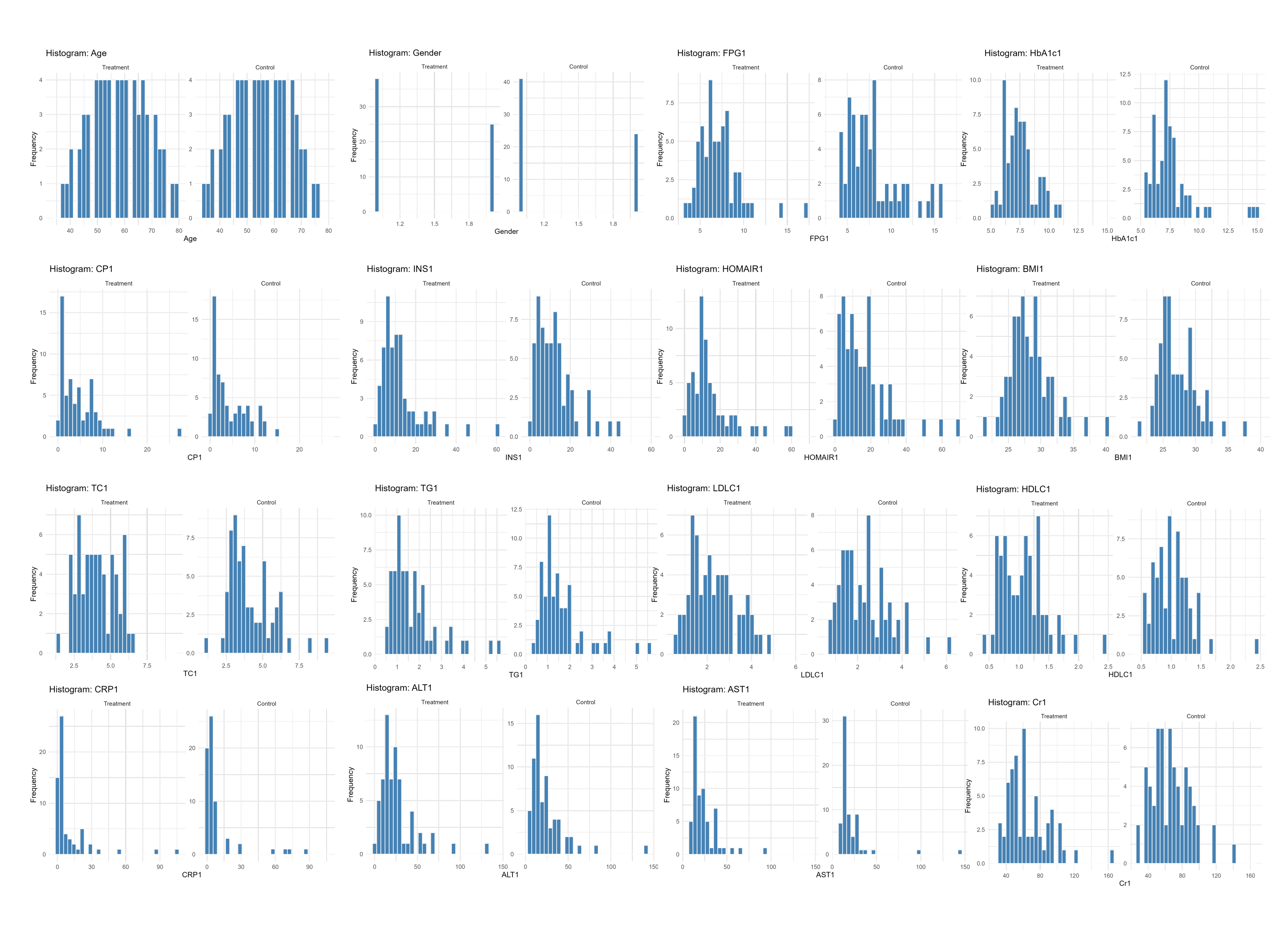


Figure S3 shows histogram of selected baseline variables with fitted normal density curves. The plots indicate approximate distributional patterns of the baseline data.

## Figure S5. Normal Q-Q plots of baseline variables.


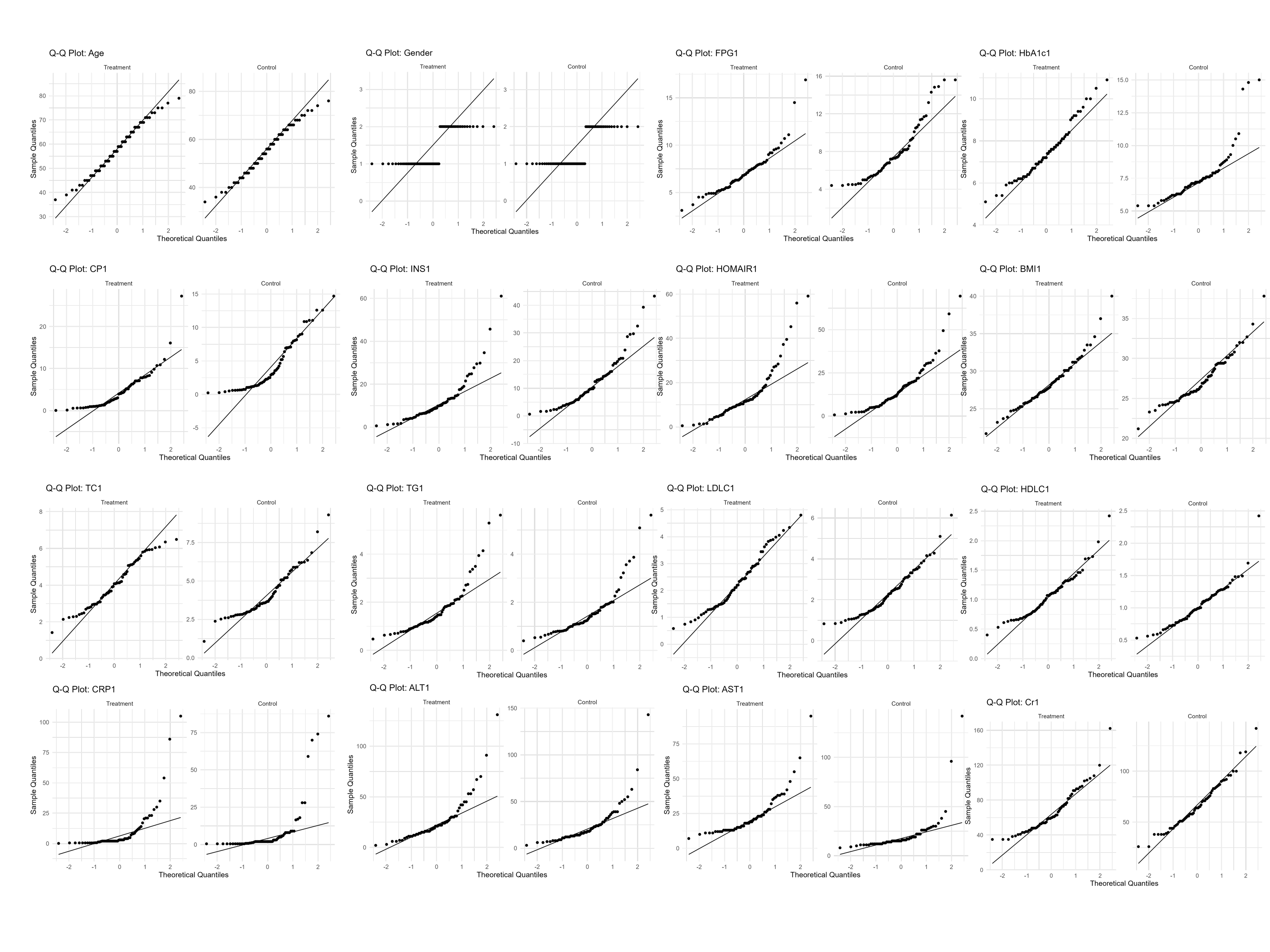


Normal Q–Q plots for the baseline variables. Data points lying close to the diagonal line suggest approximate normality, supporting the use of parametric statistical methods in subsequent analyses.
