# Supplementary material for: Randomized trial and multi-omics, machine learning–based mechanistic exploration of daixie decoction granules in type 2 diabetes
Source: Front Pharmacol. 2026 Jan 5;16:1723584. doi: 10.3389/fphar.2025.1723584 (PMC12812742; doi:10.3389/fphar.2025.1723584)
Supplement: Supplementary file 1 [file DataSheet1.zip › Supplementary Material3.docx]

## Supplementary Material 3

**Part1. R language codes for 7 machine learning screening genes**

# ======================= 0. Set working directory ===========================

setwd("XXXXXXXX")

# ======================= 1. Load packages ================================

library(caret)

library(DALEX)

library(ggplot2)

library(randomForest)

library(kernlab)

library(xgboost)

library(pROC)

library(rpart)

library(glmnet)

library(nnet)

library(auditor)

library(precrec)

library(dplyr)

set.seed(123)

# ======================= 2. Input data ===============================

expr_file <- "MATRIX.csv" # First row = samples, first column = gene names

design_file <- "design.txt"

expr <- read.csv(expr_file, row.names = 1, check.names = FALSE)

design <- read.table(design_file, sep="\t", header=TRUE, stringsAsFactors=FALSE)

# Transpose: rows = samples, columns = genes

expr_t <- t(expr)

expr_t <- as.data.frame(expr_t)

expr_t$id <- rownames(expr_t)

# Merge phenotype

data <- merge(design, expr_t, by="id")

rownames(data) <- data$id

data$id <- NULL

# Ensure Type is factor with desired level order

data$Type <- factor(data$Type, levels = c("control", "treat"))

# ======================= 3. Train/test split =========================

inTrain <- createDataPartition(y=data$Type, p=0.7, list=FALSE)

train <- data[inTrain, ]

test <- data[-inTrain, ]

# Align factor levels

train$Type <- factor(train$Type, levels = levels(data$Type))

test$Type <- factor(test$Type, levels = levels(data$Type))

# ======================= 4. Cross-validation setup ===========================

control <- trainControl(method="repeatedcv",

number=5,

repeats=5,

savePredictions=TRUE,

classProbs=TRUE,

summaryFunction=twoClassSummary)

# ======================= 5. Model list (seven algorithms) ====================

models <- list(

knn = "knn", # KNN

nnet = "nnet", # NNET

svm = "svmRadial", # SVM

glm = "glm", # GLM

dt = "rpart", # Decision tree

rf = "rf", # Random forest

lasso = "glmnet" # LASSO

)

# ======================= 6. Train models ===============================

trained_models <- list()

for (m in names(models)) {

cat("Training model:", m, "\n")

trained_models[[m]] <- train(

Type ~ .,

data=train,

method=models[[m]],

trControl=control,

metric="ROC"

)

}

# ======================= 7. Test-set prediction ============================

yTest <- ifelse(test$Type=="control", 0, 1)

pred_probs <- list()

pred_classes <- list()

for (m in names(trained_models)) {

pred_probs[[m]] <- predict(trained_models[[m]], test, type="prob")[,2]

pred_classes[[m]] <- predict(trained_models[[m]], test)

}

# ======================= 8. Performance metrics ======================

metrics <- data.frame()

for (m in names(trained_models)) {

pred_classes[[m]] <- factor(pred_classes[[m]], levels = levels(test$Type))

cm <- confusionMatrix(pred_classes[[m]], test$Type)

acc <- cm$overall["Accuracy"]

sens <- cm$byClass["Sensitivity"]

spec <- cm$byClass["Specificity"]

f1 <- cm$byClass["F1"]

metrics <- rbind(metrics,

data.frame(Model=m,

Accuracy=acc,

Sensitivity=sens,

Specificity=spec,

F1=f1))

}

write.csv(metrics, "Model_metrics.csv", row.names=FALSE)

print(metrics)

# ======================= 9. ROC curves ===============================

pdf("ROC_all_models.pdf", width=7, height=7)

colors <- rainbow(length(trained_models))

i <- 1

for (m in names(trained_models)) {

roc_obj <- roc(yTest, pred_probs[[m]])

if (i==1) {

plot(roc_obj, col=colors[i], main="ROC Curves",

xlab="False Positive Rate", ylab="True Positive Rate", lwd=2)

} else {

plot(roc_obj, col=colors[i], add=TRUE, lwd=2)

}

i <- i+1

}

legend("bottomright",

legend=paste(names(trained_models),

"AUC=", sapply(pred_probs, function(p) round(roc(yTest,p)$auc,3))),

col=colors, lwd=2)

dev.off()

# ======================= 10. PRC curves ==============================

mmdata <- mmdata(pred_probs, yTest, modnames = names(trained_models))

mmcurves <- evalmod(mmdata)

pdf("PRC_all_models.pdf", width=7, height=7)

autoplot(mmcurves, "PRC") +

ggtitle("Precision–Recall Curves") +

xlab("Recall") + ylab("Precision")

dev.off()

# ======================= 11. Feature importance =======================

library(caret)

models_order <- names(trained_models)

get_top10_imp <- function(mod) {

vi <- varImp(mod, scale = TRUE)$importance

num_cols <- sapply(vi, is.numeric)

vi_num <- vi[, num_cols, drop = FALSE]

imp_col <- names(vi_num)[which.max(apply(vi_num, 2, var))]

imp <- vi_num[, imp_col, drop = FALSE]

colnames(imp) <- "Importance"

imp$Gene <- rownames(imp)

imp <- imp[order(-imp$Importance), ]

head(imp, 10)

}

cols <- c(

"#A6CEE3", "#B2DF8A", "#FB9A99", "#FDBF6F",

"#CAB2D6", "#FFFF99", "#1F78B4", "#AED6F1",

"#F4ECF7", "#D5F5E3"

)

pdf("Variable_importance_multiModels_softColor.pdf", width = 8, height = 10)

n_mod <- length(models_order)

n_row <- ceiling(n_mod / 2)

par(mfrow = c(n_row, 2), mar = c(4, 10, 3, 2))

for (m in models_order) {

top10 <- get_top10_imp(trained_models[[m]])

top10 <- top10[order(top10$Importance), ]

barplot(

top10$Importance,

names.arg = top10$Gene,

horiz = TRUE,

las = 1,

col = cols,

border = NA,

main = paste("Variable Importance -", m),

xlab = "Importance",

cex.names = 0.8

)

}

dev.off()

# ======================= 12. Compute residuals =======================

get_prob <- function(mod) {

p <- predict(mod, newdata = test, type = "prob")

p[, 2]

}

residuals_list <- list()

for (m in names(trained_models)) {

prob <- get_prob(trained_models[[m]])

resid <- yTest - prob

residuals_list[[m]] <- resid

}

# ======================= Residual CDF (two-sided) =======================

tsecdf_data <- function(resid) {

r <- sort(resid)

n <- length(r)

F <- seq_len(n) / n

ts <- ifelse(r <= 0, F, 1 - F)

data.frame(resid = r, tsecdf = 2 * ts)

}

pdf("Residuals_CDF_allModels_simple.pdf", width = 7, height = 5)

cols <- rainbow(length(residuals_list))

i <- 1

for (m in names(residuals_list)) {

dat <- tsecdf_data(residuals_list[[m]])

if (i == 1) {

plot(dat$resid, dat$tsecdf, type = "l", lwd = 2,

col = cols[i],

xlab = "Residual (y - p)",

ylab = "Two-sided ECDF",

main = "Residuals CDF - All Models")

} else {

lines(dat$resid, dat$tsecdf, col = cols[i], lwd = 2)

}

i <- i + 1

}

legend("topright", legend = names(residuals_list),

col = cols, lwd = 2, cex = 0.8)

dev.off()

# ======================= Residual density =======================

pdf("Residuals_Density_allModels_simple.pdf", width = 7, height = 5)

cols <- rainbow(length(residuals_list))

i <- 1

for (m in names(residuals_list)) {

dens <- density(residuals_list[[m]], na.rm = TRUE)

if (i == 1) {

plot(dens,

main = "Residuals Density - All Models",

xlab = "Residual (y - p)",

ylab = "Density",

lwd = 2,

col = cols[i])

} else {

lines(dens, col = cols[i], lwd = 2)

}

i <- i + 1

}

legend("topright", legend = names(residuals_list),

col = cols, lwd = 2, cex = 0.8)

dev.off()

**Part2. Performance of seven machine-learning models in the internal test set**

**Table S1. Performance of seven machine-learning models in the internal test set**

| Model | AUC | Accuracy | Sensitivity | Specificity | F1 |
| --- | --- | --- | --- | --- | --- |
| knn | 0.80 | 0.67 | 0.80 | 0.55 | 0.70 |
| nnet | 0.71 | 0.62 | 0.50 | 0.73 | 0.56 |
| svm | 0.79 | 0.57 | 0.80 | 0.36 | 0.64 |
| glm | 0.56 | 0.48 | 0.60 | 0.36 | 0.52 |
| dt | 0.56 | 0.43 | 0.40 | 0.45 | 0.40 |
| rf | 0.84 | 0.67 | 0.60 | 0.73 | 0.63 |
| lasso | 0.76 | 0.71 | 0.80 | 0.64 | 0.73 |

**Part3. Literature evidence for core genes.**

**Table S2. Literature evidence supporting the association between prioritized targets and T2DM-related pathways.**

| **Gene** | **Main axis / function** | **Evidence type** | **Key findings related to T2DM** | **Representative references** |
| --- | --- | --- | --- | --- |
| **P2RX7** | NLRP3 inflammasome activation, IL-1β release | Experimental / Clinical | ATP-driven P2X7 receptors promote NLRP3 inflammasome overexpression and IL-1β release in diabetic conditions. P2X7/NLRP3 pathway amplifies inflammation via ATP feedback loop in hyperglycemia-induced retinal endothelial cells. Excessive P2X7-NLRP3 activation leads to increased inflammatory cytokine release in diabetes. | 1. Kong H, Zhao H, Chen T, Song Y, Cui Y. Targeted P2X7/NLRP3 signaling pathway against inflammation, apoptosis, and pyroptosis of retinal endothelial cells in diabetic retinopathy. Cell Death Dis. 2022;13(4):336. Published 2022 Apr 12. doi:10.1038/s41419-022-04786-w 2. Karmakar M, Katsnelson MA, Dubyak GR, Pearlman E. Neutrophil P2X7 receptors mediate NLRP3 inflammasome-dependent IL-1β secretion in response to ATP. Nat Commun. 2016;7:10555. Published 2016 Feb 15. doi:10.1038/ncomms10555 |
| **IL1B** | Pro-inflammatory cytokine, β-cell function | Clinical / Experimental | High glucose induces IL-1β production in pancreatic β-cells leading to impaired insulin secretion and apoptosis. IL-1β-producing β-cells observed in T2DM patients but not controls. Blocking IL-1 with anakinra improved glycemia and β-cell function in clinical trials. Increased IL-1β mRNA expression in β-cells of T2DM individuals. | 1. Maedler K, Sergeev P, Ris F, et al. Glucose-induced beta cell production of IL-1beta contributes to glucotoxicity in human pancreatic islets. J Clin Invest. 2002 Sep;110(6):851-60. doi: 10.1172/JCI15318. 2. Maedler K, Sergeev P, Ris F, et al. Glucose-induced β cell production of IL-1β contributes to glucotoxicity in human pancreatic islets. J Clin Invest. 2017;127(4):1589. doi:10.1172/JCI92172 |
| **PTPN1** | Negative regulator of insulin signaling | Experimental / Genetic | PTPN1 encodes PTP1B which dephosphorylates insulin receptor, negatively regulating insulin signaling. PTPN1 gene polymorphisms associated with T2DM susceptibility (OR ~1.3, PAR 17-20%). Whole-body PTP1B knockout mice show enhanced insulin sensitivity and resistance to diet-induced obesity. PTPN1 located in chromosome 20q13 region linked to T2DM. | 1. Bento JL, Palmer ND, Mychaleckyj JC, et al. Association of protein tyrosine phosphatase 1B gene polymorphisms with type 2 diabetes. Diabetes. 2004;53(11):3007-3012. doi:10.2337/diabetes.53.11.3007 2. Cheyssac C, Lecoeur C, Dechaume A, et al. Analysis of common PTPN1 gene variants in type 2 diabetes, obesity and associated phenotypes in the French population. BMC Med Genet. 2006;7:44. Published 2006 May 5. doi:10.1186/1471-2350-7-44 |
| **AKT2** | Insulin signaling, glucose uptake | Genetic / Experimental | AKT2 critical for insulin-stimulated glucose uptake in skeletal muscle. Loss-of-function mutation (R274H) causes severe insulin resistance and diabetes in humans. Akt2 knockout mice display insulin resistance and diabetic phenotype. Insulin-stimulated Akt2 activity decreased in obese insulin-resistant individuals. AKT2 phosphorylates AS160 promoting GLUT4 translocation. | 1. Han Cho et al. Insulin Resistance and a Diabetes Mellitus-Like Syndrome in Mice Lacking the Protein Kinase Akt2 (PKBβ). Science292,1728-1731(2001). DOI:10.1126/science.292.5522.1728 2. Vogan, K. AKT2 mutations and hypoglycemia. Nat Genet 43, 1178 (2011). <https://doi.org/10.1038/ng.1024> |
| **CD38** | NAD⁺ / Ca²⁺ signaling, β-cell function | Experimental | CD38 is major NAD-consuming enzyme; hyperglycemia activates CD38 reducing NAD⁺/NADH ratio promoting metabolic dysfunction. CD38 deficiency suppressed insulin signaling and increased apoptosis in pancreatic islets. CD38-mediated Ca²⁺ signaling contributes to glucagon-induced hepatic gluconeogenesis. CD38 inhibition restores NAD⁺ levels and improves metabolic parameters in obesity/diabetes models. | 1. Escande C, Nin V, Price NL, et al. Flavonoid apigenin is an inhibitor of the NAD+ ase CD38: implications for cellular NAD+ metabolism, protein acetylation, and treatment of metabolic syndrome. Diabetes. 2013;62(4):1084-1093. doi:10.2337/db12-1139 2. Camacho-Pereira J, Tarragó MG, et al. CD38 Dictates Age-Related NAD Decline and Mitochondrial Dysfunction through an SIRT3-Dependent Mechanism. Cell Metab. 2016 Jun 14;23(6):1127-1139. doi: 10.1016/j.cmet.2016.05.006. PMID: 27304511; PMCID: PMC4911708. |
| **NFE2L2** | Antioxidant response, oxidative stress | Experimental / Clinical | NFE2L2 encodes NRF2, master regulator of antioxidant defense. NRF2 pathway dysregulated in diabetes with impaired nuclear translocation. Activation of NRF2 reduces oxidative stress and improves diabetic complications. NRF2 polymorphism rs6721961 associated with T2DM risk and impaired β-cell function. Defective NRF2-dependent redox signaling contributes to microvascular dysfunction in T2DM. | 1. Uruno A, Furusawa Y, Yagishita Y, et al. The Keap1-Nrf2 system prevents onset of diabetes mellitus. Mol Cell Biol. 2013;33(15):2996-3010. doi:10.1128/MCB.00225-13 2. Uruno A, Yagishita Y, Yamamoto M. The Keap1-Nrf2 system and diabetes mellitus. Arch Biochem Biophys. 2015;566:76-84. doi:10.1016/j.abb.2014.12.012 |
| **NOS3** | Endothelial NO production, vascular function | Genetic / Clinical | NOS3 encodes eNOS; polymorphisms (G894T, T-786C, 27bp-VNTR) associated with T2DM and insulin resistance. Impaired eNOS function contributes to endothelial dysfunction and diabetic vascular complications. eNOS deficiency in diabetic mice causes severe nephropathy with increased albuminuria. Decreased NO bioavailability in T2DM leads to vascular resistance (~13-94% reduction in endothelium-dependent relaxation). | 1. Wang D, Liu L, Zhang C, Lu W, Wu F, He X. Evaluation of Association Studies and Meta-Analyses of eNOS Polymorphisms in Type 2 Diabetes Mellitus Risk. Front Genet. 2022;13:887415. Published 2022 Jun 27. doi:10.3389/fgene.2022.887415 2. Monti LD, Barlassina C, Citterio L, et al. Endothelial nitric oxide synthase polymorphisms are associated with type 2 diabetes and the insulin resistance syndrome. Diabetes. 2003;52(5):1270-1275. doi:10.2337/diabetes.52.5.1270 |
| **MERTK** | Efferocytosis, inflammation resolution | Experimental | MerTK receptor critical for clearance of apoptotic cells (efferocytosis). Hyperglycemia decreases MerTK expression on macrophages impairing efferocytosis in diabetes. MerTK deficiency promotes atherosclerotic plaque necrosis and inflammation. Restoration of MerTK enhances efferocytosis and attenuates atherosclerosis in diabetic models. MerTK engagement promotes pro-resolving mediator biosynthesis. | 1. Qiu S, Liu J, Chen J, et al. Targeted delivery of MerTK protein via cell membrane engineered nanoparticle enhances efferocytosis and attenuates atherosclerosis in diabetic ApoE-/- Mice. J Nanobiotechnology. 2024;22(1):178. Published 2024 Apr 13. doi:10.1186/s12951-024-02463-y 2. Thorp E, Cui D, Schrijvers DM, Kuriakose G, Tabas I. Mertk receptor mutation reduces efferocytosis efficiency and promotes apoptotic cell accumulation and plaque necrosis in atherosclerotic lesions of apoe-/- mice. Arterioscler Thromb Vasc Biol. 2008;28(8):1421-1428. doi:10.1161/ATVBAHA.108.167197 |
